# NGF-Induced Upregulation of CGRP in Orofacial Pain Induced by Tooth Movement Is Dependent on Atp6v0a1 and Vesicle Release

**DOI:** 10.3390/ijms231911440

**Published:** 2022-09-28

**Authors:** Tianjin Tao, Yi Liu, Jingqi Zhang, Wenli Lai, Hu Long

**Affiliations:** State Key Laboratory of Oral Diseases, National Clinical Research Center for Oral Diseases, Sichuan University, Chengdu 610041, China

**Keywords:** orofacial pain, trigeminal ganglia, trigeminal subnucleus caudalis, lentivirus, NGF, Atp6v0a1, CGRP

## Abstract

The nerve growth factor (NGF) and calcitonin gene-related peptide (CGRP) play a crucial role in the regulation of orofacial pain. It has been demonstrated that CGRP increases orofacial pain induced by NGF. V-type proton ATPase subunit an isoform 1 (Atp6v0a1) is involved in the exocytosis pathway, especially in vesicular transport in neurons. The objective was to examine the role of Atp6v0a1 in NGF-induced upregulation of CGRP in orofacial pain induced by experimental tooth movement. Orofacial pain was elicited by ligating closed-coil springs between incisors and molars in Sprague–Dawley rats. Gene and protein expression levels were determined through real-time polymerase chain reaction, immunostaining, and fluorescence in situ hybridization. Lentivirus vectors carrying Atp6v0a1 shRNA were used to knockdown the expression of Atp6v0a1 in TG and SH-SY5Y neurons. The release of vesicles in SH-SY5Y neurons was observed by using fluorescence dye FM1-43, and the release of CGRP was detected by Enzyme-Linked Immunosorbent Assy. Orofacial pain was evaluated through the rat grimace scale. Our results revealed that intraganglionic administration of NGF and Atp6v0a1 shRNA upregulated and downregulated CGRP in trigeminal ganglia (TG) and trigeminal subnucleus caudalis (Vc), respectively, and the orofacial pain was also exacerbated and alleviated, respectively, following administration of NGF and Atp6v0a1 shRNA. Besides, intraganglionic administration of NGF simultaneously caused the downregulation of Atp6v0a1 in TG. Moreover, the release of vesicles and CGRP in SH-SY5Y neurons was interfered by NGF and Atp6v0a1 shRNA. In conclusion, in the orofacial pain induced by experimental tooth movement, NGF induced the upregulation of CGRP in TG and Vc, and this process is dependent on Atp6v0a1 and vesicle release, suggesting that they are involved in the transmission of nociceptive information in orofacial pain.

## 1. Introduction

With a prevalence of 16% among the general population [1], orofacial pain is a constellation of painful conditions in the orofacial regions, including temporomandibular joint disorders, trigeminal neuralgia, headaches, and orthodontic pain [2,3]. These painful conditions pose significant burdens to those who suffer from orofacial pain [4]. Several modalities have been used to alleviate orofacial pain, such as non-steroid anti-inflammatory drugs (NSAIDS), surgical approaches, and behavioral therapy [5,6]. Unfortunately, to date, no truly effective or clinically validated pain-relief treatment is available [7].

This is partly attributed to a poor understanding of the underlying mechanisms for orofacial pain, and research that unravels the mechanisms of orofacial pain and offers new insights for pain-relieving is justifiable.

Evidence suggests that orofacial pain signals are first received by peripheral sensory terminals, transmitted to trigeminal ganglion (TG), relayed in the trigeminal subnucleus caudalis (Vc), and finally projected through the thalamus to the sensory cortex [8]. After being activated by orofacial pain stimuli, neurons in TG release abundant neuropeptides to the periphery, thereby facilitating inflammatory response and promoting pain transmission [8]. Therefore, the TG and the Vc are cardinal relay points in the transmission of pain signals.

As an important protein in the process of neurogenesis and neuron growth, nerve growth factor (NGF) plays an important role in orofacial pain modulation [9,10]. Our previous study revealed that the administration of NGF into trigeminal ganglia could exacerbate orofacial pain [11]. Recent studies have also proved that NGF has been associated with the upregulation of CGRP, leading to neuroinflammatory pain response [2,12]. This suggests that NGF and CGRP are cardinal molecules in the modulation of orofacial pain. It is well documented that TG could receive signals from peripheral nerve terminals and transmit them to the Vc. Our previous study revealed that CGRP in Vc may derive from the nerve extending from trigeminal neurons [13]. However, to the best of our knowledge, few studies have explored the mechanism of CGRP transport and release to the central terminal after synthesis in trigeminal ganglion neurons.

Moreover, signal transduction between neurons is accomplished by synaptic vesicle exocytosis, which relies on vacuolar proton-pumping ATPase (V-ATPase) [14]. Studies have revealed that the membrane fusion process in which the V-type proton ATPase subunit an isoform 1 (Atp6v0a1) is partially involved plays an important role in the exocytosis pathway, especially in vesicular transport in neurons [14]. Thus, we hypothesized that following NGF-mediated orofacial pain induced by experimental tooth movement, CGRP synthesized in TG might transport to Vc through vesicle transport, and this process is dependent on Atp6v0a1. In this study, we aimed to explore the mechanism underlying NGF-CGRP-mediated orofacial pain.

## 2. Results

### 2.1. NGF Participated in the Regulation of Orofacial Pain through Upregulating CGRP in Trigeminal Ganglia

**Figure 1:**
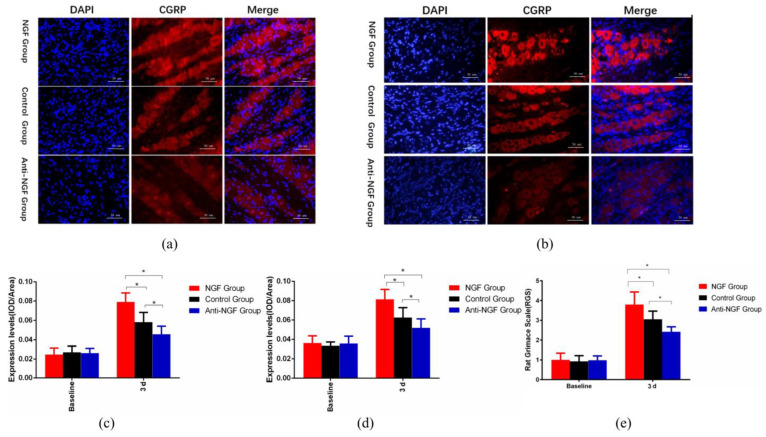
NGF exacerbates orofacial pain by upregulating the expression of CGRP in trigeminal neurons. (**a**,**d**) The effects of NGF and Anti NGF on the expression of CGRP in trigeminal ganglia in vivo (**a**) Immunofluorescence (×400). (**b**) Fluorescence in situ hybridization (×400). (**c**) Quantification of immunofluorescence. (**d**) Quantification of fluorescence in situ hybridization. (**e**) The effects of NGF and Anti NGF on orofacial pain (Data are presented as mean ± SD, *n* = 6. *: *p* < 0.05).

As depicted in Figure 1a–d, immunofluorescence (Figure 1a,c) and fluorescence in situ hybridization (Figure 1b,d) revealed that intraganglionic administration of NGF could upregulate CGRP expression in TG (*p* < 0.05), while the administration of anti-NGF could downregulate CGRP expression (*p* < 0.05). As displayed in Figure 1e, our results reveal that the orofacial pain level was significantly elevated in the NGF group than in the control group (*p* < 0.05), and compared with the control group, the orofacial pain level was lower in anti-NGF group (*p* < 0.05).

### 2.2. NGF-Induced Regulation of CGRP in Trigeminal Subnucleus Caudalis (Vc)

**Figure 2:**
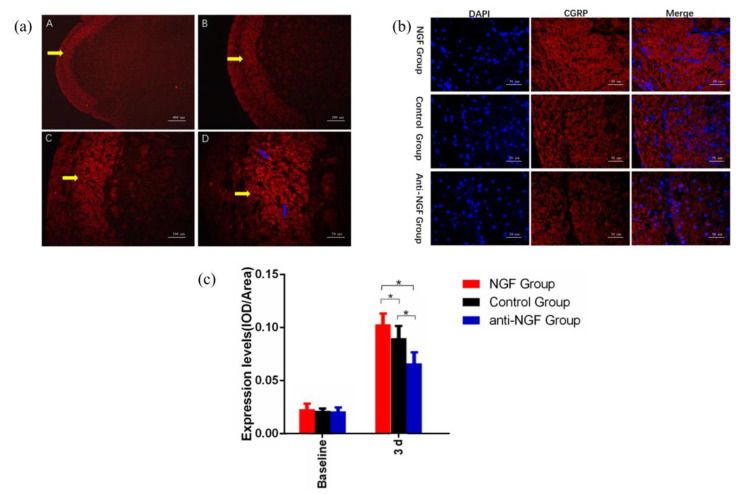
The expression of CGRP in Trigeminal Subnucleus Caudalis (Vc) in the orofacial pain model in different groups. (**a**) The yellow arrow indicates the position of the superficial layer of Vc, and the blue arrow indicates the CGRP-positive nerve fibers (A: ×50; B: ×100; C: ×200; D: ×400). (**b**) Immunofluorescence detection of CGRP expression in the superficial layer of Vc in NGF group, Control group, and Anti-NGF group at 3d (×400). (**c**) The comparison of CGRP expression levels between NGF group, the Control group, and Anti-NGF group (Data are presented as mean ± SD, *n* = 6. *: *p* < 0.05).

As presented in Figure 2, the expression level of CGRP in Vc was upregulated at 3d (*p* < 0.05). At the same time, we found that compared with the control group, the expression level of CGRP was higher in NGF group, while that for anti-NGF group was lower at 3d (*p* < 0.05), indicating that intraganglionic administration of NGF and anti-NGF into TG can regulate the expression of CGRP in Vc.

### 2.3. NGF Upregulated CGRP Is Independent on Atp6v0a1 in TG

**Figure 3:**
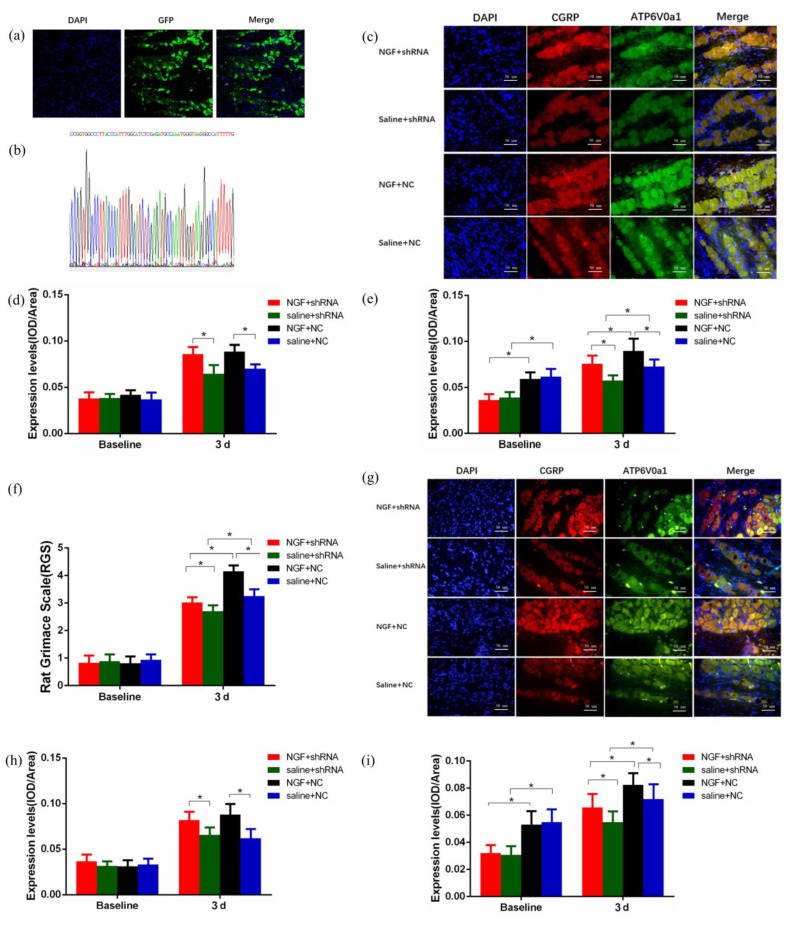
The Effects of NGF and Atp6v0a1on CGRP Expression in TG. (**a**) Lentivirus vector was successfully transduced into trigeminal neurons in vivo. (**b**) DNA sequencing confirmed the successful recombination of Atp6v0a1 short hairpin RNA (shRNA) sequence and virus vector. (**c**–**e**) Comparison of average optical density of the expression levels of CGRP and Atp6v0a1 proteins among Saline + NC group, NGF + NC group, Saline + shRNA group, and NGF + shRNA group in vivo at 3d (**c**) immunofluorescence imaging (×400). (**d**) Quantification of fluorescence for CGRP protein. (**e**) quantification of fluorescence for Atp6v0a1 protein. (**f**) Comparison of orofacial pain level among Saline + NC group, NGF + NC group, Saline + shRNA group, and NGF + shRNA group through rat grimace scale (RGS). (**g**–**i**) Comparison of average optical density of the expression levels of CGRP and Atp6v0a1 mRNA among Saline + NC group, NGF + NC group, Saline + shRNA group, and NGF + shRNA group in trigeminal ganglion at 3d. (**g**) Fluorescence in situ hybridization imaging (×400). (**h**) Quantification of fluorescence for CGRP mRNA. (**i**) Quantification of fluorescence for Atp6v0a1 mRNA (Data are presented as mean ± SD, *n* = 6. *: *p* < 0.05).

As shown in Figure 3, the lentivirus vector was successfully transduced into trigeminal neurons by intraganglionic administration (Figure 3a). Moreover, we confirmed the sequences of the viral vectors carrying Atp6v0a1 shRNA through DNA sequencing (Figure 3b). The results demonstrated that lentivirus vectors were detected in TG 10d after transduction. The immunofluorescence (Figure 3c–e) and fluorescence in situ hybridization (Figure 3g–i) results are displayed in Figure 3. CGRP is mainly expressed in the cytoplasm of TG (Figure 3c). Semi-quantitative analysis of the expression levels of CGRP in TG (Figure 3d) demonstrated that, compared with baseline, the expression level of CGRP protein in TG at 3d significantly increased for all the groups (*p* < 0.05). At the same time, we found that the expression level of CGRP protein in NGF + Atp6v0a1 shRNA group was significantly higher than that of saline + Atp6v0a1 shRNA group (*p* < 0.05) and compared with saline + NC group, CGRP expression in NGF + NC group elevated significantly (*p* < 0.05). Similar results for CGRP mRNA expression levels by fluorescence in situ hybridization (FISH) were shown in Figure 3g,h. Similarly, the analysis of the protein and mRNA expression levels of Atp6v0a1 (Figure 3c,e,g,i) indicates that Atp6v0a1 expression levels were successfully interfered by shRNA, and intraganglionic administration of NGF could upregulate Atp6v0a1 expression in TG (*p* < 0.05).

### 2.4. The Expression of CGRP in Vc Is Dependent on NGF and Atp6v0a1

**Figure 4:**
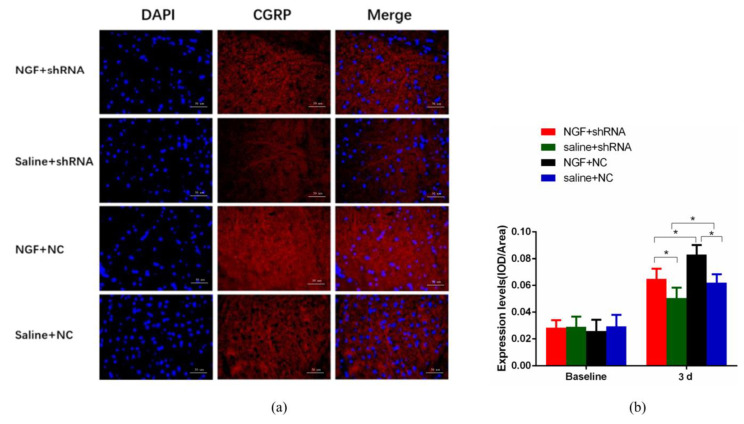
The expression of CGRP in Trigeminal Subnucleus Caudalis (Vc) for Saline + NC group, NGF + NC group, Saline + Atp6v0a1 shRNA group, and NGF + Atp6v0a1 shRNA group. (**a**) Immunofluorescence detection of CGRP expression in the superficial layer at 3d (×400). (**b**) The comparison of CGRP expression level among Saline + NC group, NGF + NC group, Saline + Atp6v0a1 shRNA group, and NGF + Atp6v0a1 shRNA group (Data are presented as mean ± SD, *n* = 6. *: *p* < 0.05).

As displayed in Figure 4, semi-quantitative analysis revealed that NGF could upregulate the expression level of CGRP in Vc, while Atp6v0a1 shRNA could down-regulate the expression of CGRP. Specifically, we found that the expression level of CGRP in Vc at 3d in Saline + NC group was higher than that in NC + Atp6v0a1 shRNA group, and the difference was statistically significant (*p* < 0.05). In the NGF + Atp6v0a1 shRNA group, the expression of CGRP was significantly lower than that in the NGF + NC group (*p* < 0.05). Similarly, the expression level of CGRP in Vc at 3d in NGF + Atp6v0a1 shRNA group was significantly higher than that in Saline + Atp6v0a1 shRNA group (*p* < 0.05). Besides, in the NGF + NC group, the expression of CGRP was higher than that in Saline + NC group, and the difference was statistically significant (*p* < 0.05).

### 2.5. In Vitro NGF Modulate Synaptic Vesicle and CGRP Release from SH-SY5Y Neurons Is Dependent on Atp6v0a1

**Figure 5:**
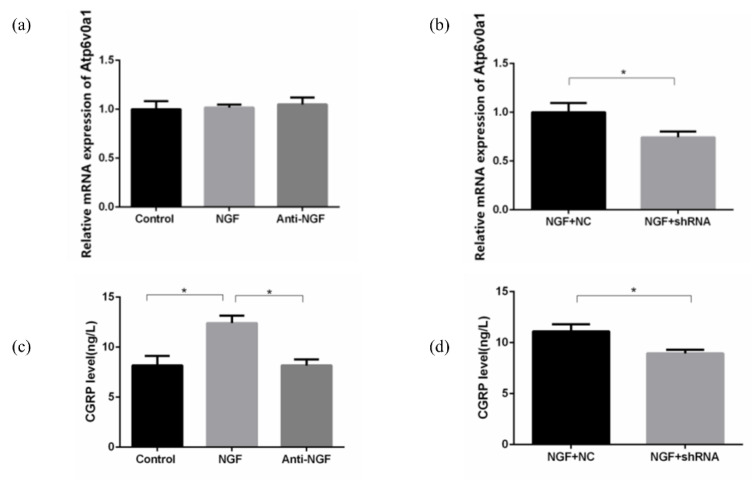
The expression level of Atp6v0a1 and CGRP in SH-SY5Y neurons for Control group, NGF group, Anti-NGF group, NGF + NC group, and NGF + Atp6v0a1 shRNA group. (**a**,**b**) Real-time polymerase chain reaction results for Atp6v0a1 expression level. (**c**,**d**) Enzyme-Linked Immunosorbent Assy (ELISA) results for CGRP expression level (Data are presented as mean ± SD. *: *p* < 0.05).

In cultured SH-SY5Y neurons, as presented in Figure 5a,b, there was no statistical difference in the expression of Atp6v0a1 induced by NGF or anti-NGF, while compared with NGF + NC group, Atp6v0a1 shRNA could significantly reduce the expression of Atp6v0a1 (*p* < 0.05).

**Figure 6:**
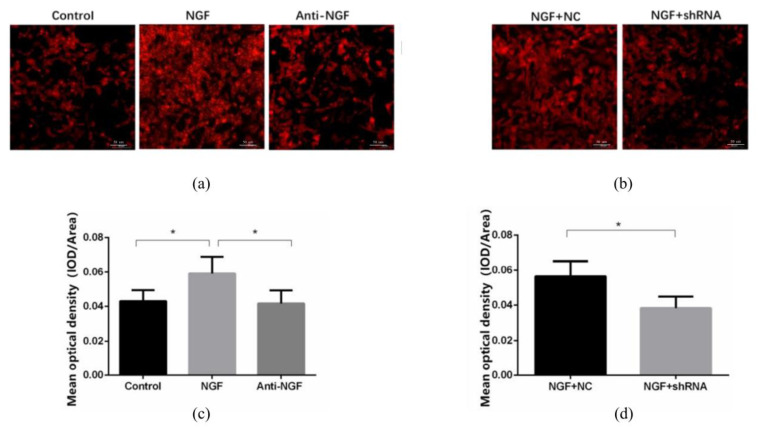
FM1-43 fluorescent staining was used to detect the vesicle release in SH-SY5Y neurons for Control group, NGF group, Anti-NGF group, NGF + NC group, and NGF + Atp6v0a1 shRNA group. (**a**,**b**) FM1-43 fluorescent staining imaging (×200). (**c**,**d**) Comparison of FM1-43 fluorescence intensity (Data are presented as mean ± SD. *: *p* < 0.05).

FM1-43 fluorescent staining was used to detect the vesicle release of SH-SY5Y neurons in each group (Figure 6). The results showed that compared with the control group and anti-NGF group, the fluorescence intensity of the NGF group was significantly increased (*p* < 0.05). Moreover, compared with the NGF + NC group, the fluorescence intensity of NGF + Atp6v0a1 shRNA group was lower, and the difference was statistically significant (*p* < 0.05).

The CGRP protein of SH-SY5Y neurons in each group was detected by Enzyme-Linked Immunosorbent Assy (ELISA). CGRP was expressed in the supernatant of SH-SY5Y neurons in each group after 72 h of drug administration. As shown in Figure 5c,d, compared with the NGF group, the expression of CGRP in the supernatant of the control group and anti-NGF group was significantly decreased (*p* < 0.05). Besides, the CGRP expression of the NGF + Atp6v0a1 shRNA group was significantly lower compared to the NGF + NC group (*p* < 0.05).

## 3. Discussion

As a 130-kDa protein, NGF is involved in nerve growth and repair [15] and plays an important part in pain regulation [16]. In the process of orofacial pain induced by experimental tooth movement, the trigeminal nerve endings of the periodontal ligament were damaged, then the signals were transmitted to TG, which activated the neurons in trigeminal ganglia and led to upregulated NGF expression in TG [17,18]. Previous studies demonstrated that local administration of NGF could elicit pain while neutralizing anti-NGF could significantly alleviate pain [19], and recent studies revealed that NGF has been associated with the upregulation of CGRP, leading to neuroinflammatory pain response [2,12]. This is consistent with our results (Figure 1 and Figure 2) that the expression levels of CGRP in TG and Vc were elevated following intraganglionic administration of NGF, and administration of anti-NGF could reduce orofacial pain and the expression of CGRP.

The signals of orofacial pain induced by tooth movement are transmitted through trigeminal ganglia (TG), relayed in the trigeminal subnucleus caudalis (Vc), and finally perceived by the sensory cortex [8,20]. Among these sensory stations, TG and Vc are of vital importance to the modulation of orofacial pain. As the first-order sensory neurons in the orofacial region, TG could receive signals from peripheral nerve terminals, and transmit it to the Vc. Our previous study revealed that CGRP in TG participates in the regulation of orofacial pain, and the knockdown of CGRP through lentivirus in TG was able to attenuate orofacial pain [2]. Moreover, CGRP release in TG and Vc was upregulated following orofacial pain induced by experimental tooth movement, and CGRP in Vc may derive from the nerve extending from trigeminal neurons [13]. However, the mechanism of CGRP transport and release to the second-order sensory neurons after synthesis in trigeminal ganglion neurons remains elusive.

It has been documented that signal transduction between neurons is accomplished by synaptic vesicle exocytosis, which relies on vacuolar proton-pumping ATPase (V-ATPase) [14]. V-ATPase is a membrane protein complex with a molecular weight of about 900-kDa, which is mainly composed of V0, V1, and accessory subunits [21,22]. V0a, with a molecular weight of 100 kDa, is the largest subunit of V0, and there are four subtypes of a1–a4 [23]. It has been shown that the membrane fusion process in which V0 is partially involved is the core process of exocytosis pathway, especially in the vesicular transport of nerve cells. Atp6v0a1, the a1 subunit of V0, plays an important role in the membrane fusion process of vesicle transport [14,24]. Based on this, we hypothesized that following NGF-mediated orofacial pain induced by experimental tooth movement, CGRP synthesized in TG might transport to Vc through vesicle transport, which is dependent on Atp6v0a1. In this study, we confirmed that the expression levels of CGRP in TG and Vc were downregulated by intraganglionic administration of Atp6v0a1 shRNA and were upregulated by NGF (Figure 3 and Figure 4). Similarly, the synaptic vesicle release and expression levels of CGRP in SH-SY5Y neurons were significantly increased by NGF, while when interfered with Atp6v0a1 shRNA, both the vesicle release and expression of CGRP were downregulated (Figure 5). This is consistent with our hypothesis that the CGRP synthesized in TG might transport to Vc through vesicle transport, and this process is dependent on Atp6v0a1.

Interestingly, in vitro experiments in SH-SY5Y neurons showed that although Atp6v0a1 shRNA could successfully interfere with the expression of Atp6v0a1, both NGF group and Aiti-NGF group failed to interfere with Atp6v0a1 expression (Figure 5). However, in animal experiments, the expression levels of Atp6v0a1 were upregulated by NGF, indicating that the expression of Atp6v0a1 was dependent on NGF (Figure 3). We speculate that the differences between in vivo experiments and in vitro experiments may be due to: (1) Although SH-SY5Y cells have been used as an in vitro model in previous studies investigating pain mechanisms [25,26,27], differences between SH-SY5Y cells and trigeminal ganglion neurons exist. (2) The response of cells in vitro is different from that in the in vivo system, and the in vitro environment cannot fully simulate the effect of NGF on TG neurons during orofacial pain induced by experimental tooth movement in rats. (3) The vesicle release process of SH-SY5Y neurons induced by NGF is only partially dependent on Atp6v0a1, and further mechanisms in-depth are needed to be explored.

In conclusion, this study investigated the molecular mechanism of orofacial pain induced by experimental tooth movement, and NGF modulates orofacial pain through regulating CGRP expression both in TG and Vc, and this process is dependent on Atp6v0a1 and vesicular transport, suggesting that these molecules are involved in the transmission of nociceptive information in orofacial pain. Considering the complex regulatory mechanisms of orofacial pain, other regulatory pathways may participate in the regulation of CGRP in TG and Vc. Therefore, further studies are needed to investigate the underlying mechanisms.

## 4. Materials and Methods

### 4.1. Animals and the Induction of Orofacial Pain

Male Sprague–Dawley rats (weighing 200 to 300 g, *n* = 120) were obtained from the Animal Experimental Center of Sichuan University. They were housed in an air-conditioned room at 21 °C with a 12-h day-night cycle and provided with standard rat chow and water ad libitum. Ethical approval for this study was acquired (WCCSIRB-2015-006).

Orofacial pain was induced by ligating closed coil springs between incisors and molars in rats to mimic orofacial pain induced by orthodontic tooth movement, and this orofacial pain rat model has been well documented by previous studies [2,6,11]. Specifically, following general anesthesia with pentobarbital sodium (50 mg/kg), rats were placed in supine positions, and the closed coil springs were stretched and activated to deliver force (40 g).

### 4.2. Evaluation of Pain through the Rat Grimace Scale

Orofacial pain was evaluated through the rat grimace scale (RGS) according to the previous studies [2,28]. Briefly, we conduct the RGS score by examining the facial expression changes in the nose, eyes, ears, and whiskers of rats.

### 4.3. Administration of Drugs

The administration of drugs into the trigeminal ganglia (TG) of rats was performed according to our previous study [29]. In brief, following general anesthesia, rats were placed in lateral recumbent positions, and micro-injection needles were injected mediocaudally between tympanic bulla and the posterior borders of the mandibular ramus. Needles were advanced by 9 mm to reach the TG, then the drug suspension (10 µL) was injected. Drugs were administered 4 h prior to the assessment of RGS. Specifically, the concentrations of NGF, anti-NGF antibody solutions were 0.1 µg/µL.

### 4.4. Construction of Lentivirus Vector

After linearization, lentivirus vectors encoding enhanced green fluorescence protein (EGFP) with ubiquitin promoter were recombined with the RNAi sequence (TGGCCCTTACCCATTTGGCAT) of Atp6v0a1. Then, vector packaging and harvesting were performed by transfection of 293T cells, and its sequence was confirmed through DNA sequencing. For rats receiving lentivirus transduction, orofacial pain was elicited 10 days following virus transduction.

### 4.5. Cell Culture of SH-SY5Y

The SH-SY5Y cell lines were routinely grown in Dulbecco’s modified Eagle’s medium (DMEM) supplemented with 10% fetal bovine serum (FBS), 1% glutamine (Gln), and 1% penicillin/streptomycin (P/S). Cells were cultured at 37 °C in a saturated humidity atmosphere containing 95% air and 5% CO_2_.

### 4.6. Immunostaining

For immunostaining, tissue samples were cryosectioned at a thickness of 10 µm and thaw-mounted on slides coated with poly-L-lysine. Following fixation for 15 min in cold propanol, samples were rinsed with PBS 3 times and incubated overnight at 4 °C with primary antibodies against CGRP (1:200; Abcam; ab36001) or Atp6v0a1 (1:500; Abcam; ab105937). Afterwards, samples were incubated with secondary antibodies at 37°C for 1 h. Fluorescence microscope (AX10 imager A2/AX10 cam HRC, Zesis) with ZEN Widefield software (Version 2012, Zesis, Oberkochen, German) were used for observation and image acquisition for all sections.

### 4.7. FM1-43 Staining

As described previously [30], synaptic vesicle was marked by fluorescent dye FM1-43, and the release of it was measured by FM1-43 fluorescence intensity. Briefly, SH-SY5Y cells were incubated with 10 μM FM1-43 dye (TS5S56, Invitrogen, Corvallis, OR, USA) for 5 min to label membranes, then stimulated with high-potassium Tyrode solution for 10 min to stimulate synaptic vesicle exocytosis. After that, cells were subjected to a 5 min recovery in the presence of 10 μM FM1-43, then extensively washed with Tyrode solution to remove excess dye. The signal was detected using a confocal microscope (FV1000; Olympus, Tokyo, Japan).

### 4.8. Enzyme-Linked Immunosorbent Assy (ELISA)

Level of CGRP was measured using commercially available ELISAs (Mlbio, Shanghai, China). The samples were incubated following the manufacturer’s instructions. To quantify CGRP levels, the optical density was measured by a microplate reader (Thermo, MuLTiSKAN MK3, Couternon, France) at 450 nm, and the concentration of CGRP was calculated based on the optical density (OD) and the standard curve.

### 4.9. Fluorescence In Situ Hybridization

Fluorescence in situ hybridization (FISH) was conducted as previously described [31]. Briefly, paraffin sections (at a thickness of 4 mm) were dewaxed and hydrated. The sections were digested at 37 °C for 10 min, followed by washing with PBS for 5 min (3×) and in distilled water (1×). Then, fixed with fixative solution at RT for 10 min, and washed with distilled water (3×). Hybridization solution (20 μL/sheet) was added dropwise and incubated at 42 °C overnight. After that, 2× SSC was washed for 5 min at 37 °C (2×), 0.5× SSC was washed for 15 min at 37 °C, and 0.2× SSC was washed for 15 min at 37 °C (2×). The blocking solution was added and placed in a 37 °C constant temperature incubator for 30 min. Then, 4′,6-diamidino-2-phenylindole (DAPI) was added dropwise and incubated in the dark for 3 min, and the images were acquired by a fluorescence microscope (AX10 imager A2/AX10 cam HRC, Zesis, Oberkochen, German). The FISH probes were CGRP (5-CACACCGCTTAGATCTGGGG-3) and Atp6v0a1 (5-TAGGCTCTTCTGCGTCCTCT-3).

### 4.10. Real-Time Polymerase Chain Reaction

For real-time PCR, by using the Takara MiniBEST Universal RNA Extraction Kit (Takara, Japan), total RNA was extracted according to the manufacturer’s protocols. Then, complementary DNA (cDNA) was reversely transcribed by using the M-MLV Kit (Promega), with the heating protocol being initial activation at 95 °C for 5 min, denaturation at 95 °C for 10 s, annealing at 60 °C for 30 s, extension at 72 °C for 20 s and amplification for 40 cycles. Real-time PCR (20 µL) was performed with the aforementioned cDNA (1 µL). The reference gene was β-actin (forward: GCTATGTTGCC CTAGACTTCGA; reverse: GATGCCACAGGATTCCATACC). The testing gene was Atp6v0a1 (forward: GATGGCGGATCCAGACTTGT; reverse: CACGTAGTCGCCAGTCACAG).

### 4.11. Statistical Analysis

The differences among different groups were analyzed by One-way ANOVA (Tukey post hoc test). Student’s *t*-test was used to analyze the comparisons between the two groups. All the statistical analyses were performed in SPSS (20.0 version, Chicago, IL, USA) and GraphPad Prism (8.0 version, San Diego, CA, USA). A *p* value < 0.05 was considered statistically significant.

## 5. Conclusions

**Figure 7:**
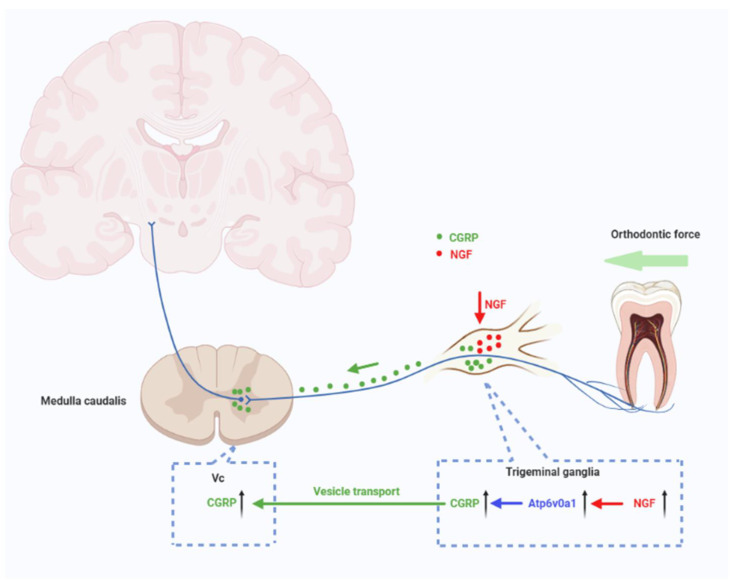
A schematic illustration depicting the mechanisms of NGF-induced upregulation of CGRP in orofacial pain induced by experimental tooth movement. The intraganglionic administration of NGF stimulates the upregulation of CGRP in TG is independent on Atp6v0a1, and the upregulation of CGRP in Vc is dependent on vesicle transport.

Therefore, CGRP expression levels were elevated both in TG and Vc, following administration of NGF in orofacial pain induced by experimental tooth movement, and this process is dependent on Atp6v0a1 and vesicle release, suggesting their involvement in the transmission of nociceptive information in orofacial pain (Figure 7). CGRP synthesized in neurons of TG could be transported to Vc through vesicle transport. However, the underlying mechanism whereby NGF and Atp6v0a1 regulate the expression of CGRP should be further elucidated.

## Data Availability

Not appliable.
